# Associations between COVID-19 Pandemic, Lockdown Measures and Human Mobility: Longitudinal Evidence from 86 Countries

**DOI:** 10.3390/ijerph19127317

**Published:** 2022-06-14

**Authors:** Md. Mokhlesur Rahman, Jean-Claude Thill

**Affiliations:** 1 Department of Urban and Regional Planning, Khulna University of Engineering & Technology, Khulna 9203, Bangladesh; mrahman.buet03@gmail.com; 2 The William States Lee College of Engineering, The University of North Carolina at Charlotte, 9201 University City Blvd, Charlotte, NC 28223, USA; 3 Department of Geography and Earth Sciences and School of Data Science, The University of North Carolina at Charlotte, 9201 University City Blvd, Charlotte, NC 28223, USA

**Keywords:** COVID-19 pandemic, lockdown measures, social distancing, human mobility, socioeconomic and institutional factor, spatial-temporal effects, SEM

## Abstract

Recognizing an urgent need to understand the dynamics of the pandemic’s severity, this longitudinal study is conducted to explore the evolution of complex relationships between the COVID-19 pandemic, lockdown measures, and social distancing patterns in a diverse set of 86 countries. Collecting data from multiple sources, a structural equation modeling (SEM) technique is applied to understand the interdependencies between independent variables, mediators, and dependent variables. Results show that lockdown and confinement measures are very effective to reduce human mobility at retail and recreation facilities, transit stations, and workplaces and encourage people to stay home and thereby control COVID-19 transmission at critical times. The study also found that national contexts rooted in socioeconomic and institutional factors influence social distancing patterns and severity of the pandemic, particularly with regard to the vulnerability of people, treatment costs, level of globalization, employment distribution, and degree of independence in society. Additionally, this study portrayed a mutual relationship between the COVID-19 pandemic and human mobility. A higher number of COVID-19 confirmed cases and deaths reduces human mobility and the countries with reduced personal mobility have experienced a deepening of the severity of the pandemic. However, the effect of mobility on pandemic severity is stronger than the effect of pandemic situations on mobility. Overall, the study displays considerable temporal changes in the relationships between independent variables, mediators, and dependent variables considering pandemic situations and lockdown regimes, which provides a critical knowledge base for future handling of pandemics. It has also accommodated some policy guidelines for the authority to control the transmission of COVID-19.

## 1. Introduction

Coronavirus disease 2019 (COVID-19) is a public health crisis that has afflicted more than 225 countries all over the world [1,2]. This highly communicable disease is expected to have lingering effects on public health, human mobility, and the environment, disrupting social relations and economic wellbeing, and transforming the social and spatial structure of the city [3,4,5,6,7,8,9,10,11]. Studies have shown that a reduction in mandatory and discretionary mobility can essentially curtail the severity of the pandemic and associated health risks [12,13]. Throughout the pandemic, various non-pharmaceutical interventions (NPIs) (e.g., lockdown and confinement, restriction on gathering and movement) have been implemented to ease the human toll significantly by reducing people’s mobility and by encouraging social distancing practices [14,15]. The spread of the pandemic has also caused people to cut their essential and non-essential movements.

Furthermore, the socioeconomic makeup and institutional structure of a country can influence pandemic severity, human mobility, and the rigor of various lockdown measures. Our previous research investigated the complex relationships between the COVID-19 pandemic, lockdown measures, and people’s social distancing and mobility behaviors [16]. However, this study was conducted at the inception of the pandemic and used mobility data for one time frame only, namely 17 April 2020. Considering the unsettled nature of these relationships over time, the current study intends to identify and analyze the evolution of the complex interplay between the COVID-19 pandemic, lockdown measures, and people’s social distancing and mobility behaviors over twelve months of the pandemic across countries globally. Focusing on temporal changes not only updates our previous work but also complements it.

In the preceding study [16], we empirically investigated the complex interplay between the COVID-19 pandemic, mobility changes in retail and recreation, transit stations, workplaces, and residential areas, and lockdown measures across a broad range of socio-spatial contexts (88 countries in total). To perform the study, data related to changes in personal mobility, socioeconomic and demographic characteristics of populations, lockdown and stay-in-place measures, and the prevalence of the COVID-19 pandemic itself were extracted from various sources (e.g., Google, UNDP, UN, BBC, Oxford University, and Worldometer). Using the framework of structural equation modeling (SEM), the direct and indirect effects of independent variables on dependent variables considering the intervening effects of mediators were explored. The early results showed that globally and at the coarse granularity of nations, lockdown measures encouraged people to maintain social distancing practices and reduced COVID-19-associated health risks. However, limited effects of pandemic severity and socioeconomic and institutional factors were observed to uphold social distancing practice. The study also reported that socioeconomic and institutional arrangements and dispositions significantly influenced the severity of the pandemic. For example, countries with a higher number of elderly, service sector employment, and higher ambiance of globalization have been the critical victims of the pandemic (e.g., USA, UK, Italy, and Spain). In contrast, social distancing measures are reasonably effective at mitigating the health risks of the pandemic.

Considering that the early effort was limited by the cross-sectional nature of the analysis, where each population sample was observed at a single point in time, the present study situates the clinical, behavioral, social, and institutional aspects of the pandemic in a broader temporal context and examines sampled countries repeatedly at different periods to detect seamlessly any changes in the variables and parameters in different phases of the pandemic. Moreover, the availability of time-series data would ensure the dynamic properties and stability of our model, which is critically important to assess the vigor of the pandemic and better empower the world for effective action under changing socio-politico-medical contexts. Despite meaningful insights from the previous study after a few months of the pandemic, a longitudinal study comprising of time series data is necessary to capture the temporal aspects of the pandemic, how they evolve and influence the mobility of the people, and how people adjust with the changing environment. This spatial-temporal data analysis is critical to mitigating the pandemic efficiently by taking data-driven decision-making [17].

Thus, this longitudinal study aims to investigate the evolution of complex relationships between the incidences of the COVID-19 pandemic, lockdown measures on populations, and their social distancing and mobility behaviors throughout the time of the pandemic from March 2020 to February 2021 across 86 countries of the world. Thanks to the integrated analytical framework of SEM, the following four research questions are formulated to understand the evolution of these intertwined relationships in different waves of the pandemic:

1. What are the impacts of various lockdown measures on reducing people’s mobility patterns and the severity of the pandemic?
2. What are the consequences of the pandemic severity on mobility patterns and, therefore, on the practice of social distancing of people?
3. What are the impacts of human mobility on the severity of the pandemic?
4. What are the effects of socioeconomic factors of domestic populations and institutional arrangements and dispositions on population mobility and on the pandemic severity?

The rest of the paper is organized as follows. Section 2 reviews the relevant strands of literature on factors of COVID-19 pandemic, lockdown and confinement measures, and human mobility, particularly in their spatio-temporal context; it is on this basis that the multidimensional conceptual framework of this study is articulated. In Section 3, we present the sources of data for the study and our analytical methods. Results of a longitudinal sequence of 24 calibrated SEM models are reported and analyzed in Section 4. The study findings are summarized and discussed in Section 5. Conclusions are drawn in Section 6.

## 2. Literature Review

A number of studies have investigated the complex relationships between the COVID-19 pandemic, lockdown measures, social distancing, and human mobility patterns. The following sub-sections summarize and discuss the associations between the conditions related to this pandemic after critically reviewing and analyzing previous studies.

### 2.1. COVID-19 Pandemic and Human Mobility Patterns

The COVID-19 pandemic has drastically changed mobility systems due to enacted lockdown and confinement measures, social distancing practices, and personal hygiene requirements, most strikingly with an increase in private travel modes (e.g., car, bicycle, walking) and a decrease in public transport (e.g., bus, train) [6,18,19,20,21,22]. Surveying 5000 urban residents in the United States, Europe, and China in April 2020, the Boston Consulting Group (BCG) investigated people’s mode choice behaviors during lockdown regimes [23]. The results shown in Table 1 illustrate that the share of some private modes (i.e., bike, scooter, walking) has increased in all three study regions due to minimal risk of COVID-19 infection. In contrast, private cars, public transit, and shared mobility options have declined, which mirrors the overall reduction in mobility in all three regions. Thus, this ongoing public health crisis has negatively and profoundly affected the travel patterns of urban residents.

**Figure 1:**
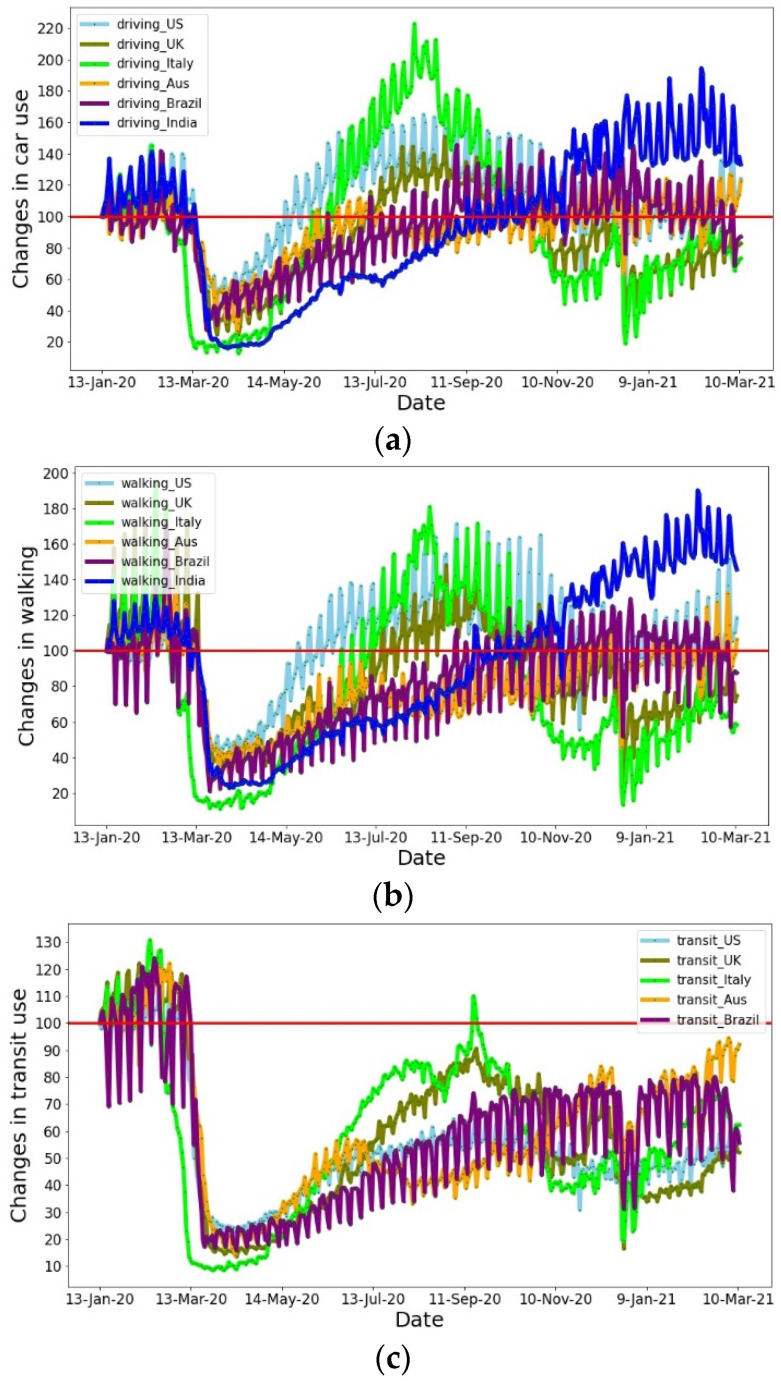
Changes in (**a**) car use, (**b**) walking, and (**c**) public transit use. 100 indicates the baseline of 13 January 2020.

Having compiled mobility data of mobile phone users by country/region/sub-region/city, Apple Inc., Cupertino, CA, USA, has estimated changes in human mobility around the world [24]. Compared to the baseline of 13 January 2020 (i.e., 100), they observed the mobility changes (i.e., time spent by the people) in car use, walking, and transit use. Figure 1 shows changes in car use, walking, and public transit use between 13 January 2020 and 11 March 2021 in six heavily affected countries, namely the United States, United Kingdom, Italy, Australia, Brazil, and India. It reveals the full extent of the mobility collapse between March and May 2020, with a deeper downturn in April. However, car and walking trips exhibited an ascending trend after May due to people’s essential travel and the low chance of infection in comparison to other modes. While public transit trips also showed an increasing trend, this remained at a level well below the baseline situation due to a higher risk of COVID-19 infection and a lower possibility to maintain social distancing. 

Many studies have investigated the impacts of COVID-19 on human mobility patterns in more detail. For example, Bucsky [19] investigated the impacts of COVID-19 and associated mobility restriction measures (e.g., national emergency, curfew, restrictions on gathering, closure of schools and colleges, border closure for non-nationals) on travel mode choice behaviors of people in Budapest, Hungary. With traffic volume data sourced from Budapest Roads Ltd., route planner application Waze, the Budapest Centre for Transport Ltd., and the local bike-sharing system, the study observed a disproportionate reduction across several transport modes. Specifically, it found a higher drop in public transit use (80%) compared to the reduction in cycling and bike sharing (23% and 2%, respectively). However, when considering the overall modal split, cycling grew to 4% in March 2020 (4%) versus 2% in 2018, as it is viewed as a safer mode with a low possibility of infection. Private car share increased dramatically from 43% in 2018 to 65% in March 2020. In contrast, public transport experienced a sharp drop during the pandemic (from 43 to 18%). Similarly, Munawar, Khan [25] calculated an 80% reduction in public transit use in Australia; thus, the transport system encountered an exceptional transformation in a short period, with a positive trend for cycling and car use and a negative trend for transit use.

Similarly, observing travel patterns of 1439 people in Switzerland, researchers in [26] estimated a 60 and 95% reduction in daily travel distance by car and public transport due to restrictive measures associated with COVID-19. They also noticed a drastic increase in cycling (i.e., a 75% increase during the lockdown period compared to 2019) due to the higher use of bicycles for non-commuting purposes (e.g., recreation). However, car travel regained slowly after the relaxation of travel restrictions. The use of public transportation was still 20% below the pre-COVID level, which indicates that many people are still working from home and either curtailed their travel altogether or their use of public transport modes as much as possible to protect themselves from infection. Besides a reduction in overall travel demand, researchers observed structural changes in human mobility patterns (e.g., reduction in long-distance travel, more clustered and local movements, changes in shortest path movement) in Germany, all of which had a considerable impact on flattening the curve [27]. Thus, the COVID-19 pandemic has affected overall transportation systems by influencing travel distance, mode choices, and changing structural attributes of travel.

Some studies also investigated how transport accessibility and human mobility may have stimulated the spreading of the COVID-19 virus. For example, researchers in [28] quantified the impacts of personal mobility on COVID-19 diffusion in Italy by developing a multiple linear regression model. The results showed a direct positive correlation of daily COVID-19 confirmed cases with trips made three weeks earlier, taking into consideration the imposed quarantine mobility restrictions of 14 days along with the incubation period of the disease; thus, human mobility is one of the critical factors that influence the diffusion of the COVID-19 pandemic [29]. In the same geographical context, Cartenì, Di Francesco [8] investigated the association between transport accessibility (i.e., rail-based accessibility) and COVID-19 cases using a multiple linear regression model. The estimated results show that transport accessibility has the highest contribution (i.e., 40%) to explaining the incidence of COVID-19 cases, which indicates that better accessibility to a certain geographic area enhances the possibility of virus diffusion. This study supported policies and strategies that restrict transportation accessibility as an effective response measure to control the COVID-19 pandemic.

Hadjidemetriou, Sasidharan [30] investigated the impacts of government interventions and human mobility on COVID-19 deaths in the United Kingdom. Using the daily digital footprint of walking, driving, and transit trips, this study found a gradual decrease in human mobility in May 2020 (i.e., 60, 80, and 60% reduction in driving, transit riding, and walking, respectively, below prior-year levels), which correlated well with the reduction of COVID-19 related deaths. It pointed to the effectiveness of travel restrictions and related non-pharmaceutical interventions (NPI), particularly while traveling on public transportation, to reduce the risk of COVID-19 infection. Otherwise, the uncontrolled movement of people would cause rapid transmission of the pandemic [31,32]. 

**Figure 2:**
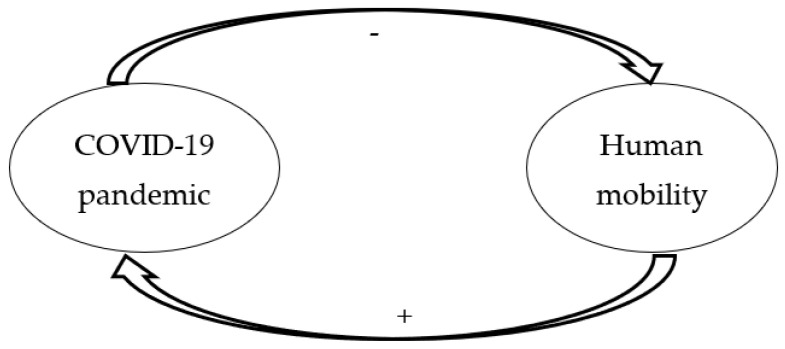
Interactions between COVID-19 and human mobility.

From the above discussion, it is conceived that COVID-19 and human mobility influence one another (Figure 2). A higher rate of virus infection reduces human mobility, while higher mobility in the population can magnify viral transmission. Thus, these two dimensions of the socio–clinical reality exhibit a bi-directional relationship.

### 2.2. Lockdown and Confinement Measures and Human Mobility

While the fear of infection and of serious health complications has gripped the state of mind of many segments of human societies, lockdown and confinement measures adopted by governments during the COVID-19 pandemic may have tangibly influenced travel patterns of people. A number of empirical studies have investigated and compared the movement of people before, during, and after the lockdown regimes to understand the impacts of NPIs on travel patterns [15,33,34,35,36,37,38,39]. 

Saha, Barman [40] investigated the impacts of lockdown measures for the COVID-19 pandemic on community mobility across India. This study used time-series trends plotting and spatial inverse distance weighted (IDW) interpolation of Google locational data to understand pre- and post-lockdown (15 February to 23 March 2020 versus 24 March to 30 April 2020) mobility changes in retail and recreation, grocery and pharmacy, parks, transit stations, and workplaces. The results indicate that lockdown and confinement measures reduced activity by 73.4, 51.2, 46.3, 66, and 56.7% in retail and recreation, grocery and pharmacy, parks, transit stations, and workplaces, respectively. In contrast, activity in residential areas increased by 23.8% since people stayed at home due to implemented travel restrictions and workplace shutdowns. The study observed a drastic change in mobility immediately just one day after (i.e., 25 March 2020) the lockdown adoption (i.e., 70.51, 60.26, 46.17, 65.6, and 60.03% reduction in activity in retail and recreation, grocery, pharmacy, parks, transit stations, workplaces, respectively, and 26.32% increase in residential areas). The study supported lockdown measures as an effective means to encourage social distancing among people to reduce COVID-19 diffusion. 

Researchers in [41] investigated the impacts of lockdown measures on travel behaviors in Thessaloniki, Greece, by comparing travel patterns before and during the pandemic. Using ordinary least squares regression and Cox proportional hazards duration models, the study reported a 50% reduction in daily trips/person due to adopted lockdown measures. It also found an increase in walking and car use and a reduction in transit trips. A sharper reduction is observed in non-commuting trips. However, overall trip duration increased substantially because people mostly travel for recreational and shopping purposes, which take much time. Similarly, some studies mentioned a 40 to 76% reduction in overall mobility in different geographical contexts of the world [19,33,35,42,43,44,45]. Thus, lockdown and confinement measures in various national contexts have a significant influence on travel behaviors, including a reduction in traffic accidents (i.e., 74.3 and 76% reduction in 14–20 February compared to 16 March–26 April 2020 and the equivalent period in 2018–2019, respectively) [46].

**Figure 3:**
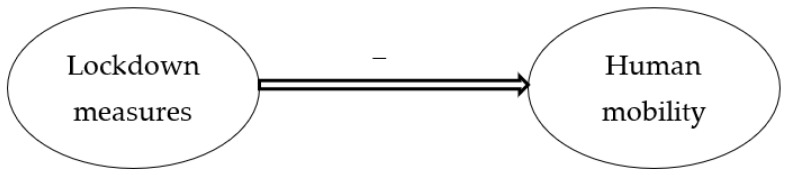
Interactions between lockdown measures and human mobility.

In Australia, Beck and Hensher [47] noticed a 50% increase in vehicle movements associated with the relaxation of travel restrictions (on 8 May 2020) that followed the initial spike in infection cases and ensuing lockdown, although vehicle movement was still 66.67% below the pre-COVID situation. Conducting a travel survey from 23 May to 15 June, they observed an increase in shopping and recreational trips and in people’s desire to socialize with friends and relatives. People are also more likely to work from home and less likely to use public transport, thus, the study suggested that government should be watchful, monitor social events, and impose social distancing measures to control viral transmission. In conclusion, the lockdown and confinement measures implemented by state authorities overall significantly influenced people’s mobility during the COVID-19 pandemic (Figure 3).

### 2.3. Lockdown and Confinement Measures and COVID-19 Transmission

As a whole, lockdown and confinement measures may play a critical role in curtailing the transmission of viruses by reducing human mobility, contacts, interactions, and social gathering. Several studies have investigated the effectiveness of lockdown and confinement measures for travel restrictions on COVID-19 transmission in cities and regions. For example, applying a data-driven epidemic model, researchers in [48] investigated the impacts of control measures (e.g., lockdown, quarantine) on COVID-19 diffusion using real-world reported cases from 24 March to 30 May 2020 in India. The prediction accuracy of the model was validated using reported confirmed cases from 1 June to 10 June 2020 with an R^2^ of 0.998. The model indicated that, after 6 weeks of the lockdown measures, confirmed cases had dropped threefold below the initial level, which sanctions the effectiveness of control measures to mitigate the severity of the pandemic. 

In the same geographical context of India, Gupta, Mohanta [49] used an SEIR-QDPA model to evaluate the impacts of statewide lockdown measures on the epidemic and to estimate exit strategies under different lockdown scenarios. With data collected in March and April 2020, they estimated the disease reproduction number to be 2.08 early on, with a drop to 1.67 on 30 March and then to 1.16 on 22 April. The decreasing trend of the reproduction number indicates the effectiveness of control measures to slow down the diffusion of COVID-19 across India. The study also found that the delay of lockdown relaxation after the first wave pushed back the start time of the second wave. In contrast, complete removal of lockdown would have increased the number of active cases despite the date of relaxation. Thus, strict lockdown measures coupled with other control measures (e.g., testing) are necessary to mitigate the severity of the pandemic [38]. 

Oum and Wang [50] determined optimal lockdowns and travel restrictions to control the pandemic in a hypothetical city or region using a traffic congestion economic model. The model indicates that when individuals engage in travel to social activities, they usually do not personalize external infection costs for other people. Consequently, this study suggested to impose travel restrictions or monetary penalties to personalize these costs to induce travel decisions and contain infectious diseases, in a fashion similar to introducing congestion pricing in the urban core to mitigate congestion. Thus, it is suggested that adopting strict measures and monetary penalties is essential to containing the pandemic in areas with a large population, population density, economic prosperity, and government-subsidized medical facilities, and a greater probability of viral transmission.

**Figure 4:**
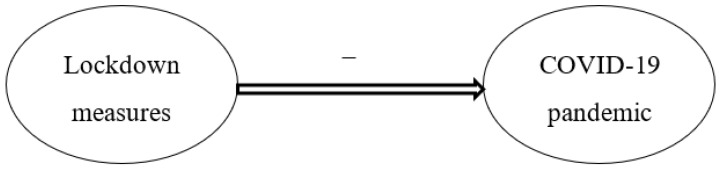
Interactions between lockdown measures and COVID-19 pandemic.

Lockdown and confinement measures tend to curtail human mobility immediately, however, they may do so differentially according to trip purposes. In addition, incubation would delay the damping of infection cases and deaths by one or two weeks [51], hence, the scope and timing of effects on health outcomes remain to be fully established. Li, Campbell [52] conducted a study in 131 countries to understand the temporal association between lockdown interventions (e.g., closure of schools and workplaces, restrictions on public gathering and movement) and COVID-19 transmission. Calculating the ratio of reproduction number (R) (i.e., the ratio of daily R and R from the previous day of implementing any lockdown measures) and applying a log-linear regression model, the study observed changes in R. The study found a reduction in R (3 to 24%) on the 28th day after any interventions compared to the last day before interventions. The study also observed an increase in R (11 to 25%) on the 28th day after the relaxation of the interventions. Thus, lockdown interventions significantly influence virus transmission, which could provide evidence to policymakers on when to introduce and relax a lockdown intervention. Similarly, by collecting data from 49 countries, researchers in [53] demonstrated that uninterrupted lockdown policies between certain dates are very effective in suppressing the COVID-19 pandemic. Thus, timely, strict, and uninterrupted lockdown measures can reduce the severity of the pandemic even in the deadly affected areas considering the lag time of around 1 to 2 weeks, as portrayed in Figure 4 [15,30,46,54,55].

### 2.4. Socioeconomic, Spatial, and Climatic Factors and COVID-19 Transmission

The socioeconomic status of people, living environments, and community structures are very important factors in viral diffusion. Much research has been conducted on the socioeconomic factors of COVID-19 transmission. These studies also investigated the temporal and spatial dependence in the associations of these factors with COVID-19 transmission. For example, Andersen, Harden [56] studied the socioeconomic and spatial determinants of the COVID-19 pandemic in the US. Collecting data from multiple sources (e.g., USAFacts, American Community Survey, Centers for Disease Control and Prevention [CDC]), the study applied a three-stage regression analysis to determine the factors that collectively influence COVID-19 cases and deaths. The results revealed that community vulnerability to the pandemic is associated with urban environments, a larger black, elderly and disabled population, and people working in production and transportation sectors with a low salary and with no sick leave benefits. Similarly, researchers in [57,58] found a higher infection and mortality rate in major cities with a higher number of elderly and a larger black population, and in less developed areas with a higher number of people with low socioeconomic background and without access to health care. 

Maiti, Zhang [59] explored the local and global associations of socioeconomic variables with COVID-19 cases and deaths using spatial regression and machine learning models. The model results indicate that ethnicity, crime rate, income, and migration have a strong correlation with the COVID-19 pandemic across US counties. The dynamic local regression model shows spatial heterogeneity in the association of socioeconomic variables with the COVID-19 pandemic. Similarly, estimated parameters in both local and global models exhibit high variability over space and time. Thus, it is effective to unravel the relationship between socioeconomic variables and COVID-19 cases and deaths by applying various models to draw more insights to control the pandemic. However, personal preventive attitudes (e.g., wearing a facemask, coughing etiquette, hand washing and sanitizing, etc.) and community preventive measures (e.g., avoiding unnecessary travel, large social gatherings, shopping, travel in crowded public transportation, and travel outside of the local area) are effective to control the severity of the pandemic [60,61].

Some studies also investigated the impacts of urban form (e.g., population density) on COVID-19 transmission. For example, Cartenì, Di Francesco [8] found that a large population and higher population density have a positive association with COVID-19 incidence. The argument here is as follows: these factors increase the possibility of infection due to increased social activity and gathering and a lower tendency to maintain social distancing. Similarly, researchers in [28,62] found that population density is positively associated with viral transmission. In contrast, researchers in [58,63] observed a negative association between density and COVID-19 incidence (i.e., areas with higher population density experience fewer COVID-19 confirmed cases and deaths). They argued that although higher density could encourage social interactions among residents, density also could lead to superior healthcare facilities (e.g., more hospitals and healthcare workers), which increase the capacity to control the pandemic. These studies advocated for compact development and walkable communities to improve public health and reduce the vulnerability of the large urban areas during any pandemic. 

Some studies have also evaluated COVID-19 incidence in different environmental and climatic conditions. For example, researchers in [64] quantified the impacts of meteorological factors (e.g., daily average temperature, relative humidity) on daily COVID-19 cases in 30 Chinese provinces. Using a generalized additive model (GAM), the study found that every 1 °C increase in average temperature with 67 to 85% relative humidity is associated with a 36 to 57% reduction in COVID-19 cases. Moreover, they noticed that every 1% increase in relative humidity reduced daily cases by 11 to 22% when the average temperatures range from 5.0 °C to 8.2 °C. However, they found spatial heterogeneity in how temperature and humidity are associated with COVID-19 incidence in Chinese provinces. Similarly, researchers in [8] found that warmer weather and the presence of a long coastline where a 30% higher temperature is observed contribute to reducing coronavirus infection. Thus, ambient temperature is inversely associated with the COVID-19 pandemic (i.e., higher temperatures reduce the number of COVID-19 cases and deaths) [28].

**Figure 5:**
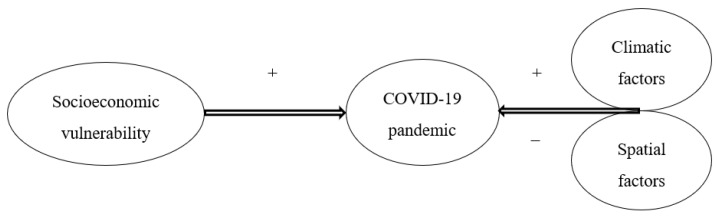
Associations between socioeconomic, spatial, and climatic factors, and the COVID-19 pandemic.

However, adjusting for city-level socioeconomic and disease control factors (i.e., lockdown), researchers in [65] found only a weak association between climatic variables (i.e., surface radiation, humidity) and COVID-19 transmission rate in 359 large cities around the world using an ordinary least squares regression. They estimated that climatic variables have less explanatory power than socioeconomic and disease control factors. Similarly, by collecting meteorological and COVID-19 incidence data from 202 locations in 8 countries and using wavelet coherence analysis, Pan, Yao [66] showed that temperature, relative humidity, wind speed, and ultraviolet (UV) radiation do not have a statistically significant association with the COVID-19 transmission rate. Moreover, validating the results using the susceptible-exposed-infectious-recovered (SEIR) model, the study also confirmed that meteorological conditions have limited impacts on COVID-19 transmission. The above discussion indicates that the socioeconomic and living environments of people have significant effects on the COVID-19 pandemic in contrast to weather conditions of that area (Figure 5). 

### 2.5. Socioeconomic and Spatial Factors and Human Mobility

Just as the COVID-19 pandemic is conditioned by socioeconomic and spatial factors, so may human mobility, including aspects of travel mode choice, travel distance, and travel time. Some studies also investigated the variations in the mobility of people from diverse sociodemographic backgrounds and geographical contexts. For example, Molloy, Schatzmann [26] observed a higher reduction in the daily travel of highly educated individuals (i.e., college or technical education) compared to less educated ones. This discrepancy became evident during and post lockdown scenarios, when individuals working in the service industry returned to work, while professionals were often not expected to do so. This study also mentioned variable daily travel across different household sizes. Single-person households experienced a lower reduction in daily travel compared to larger households due to the lesser fear of infecting other household members and greater responsibility for non-discretionary travel. Similarly, researchers in [41] found that low-income people traveled more than high-income people and the travel duration of males was higher than females. Moreover, urban residents were more likely to stay home, were unwilling to travel to the city center and other crowded places, and reduced contact with other people [62,67].

**Figure 6:**
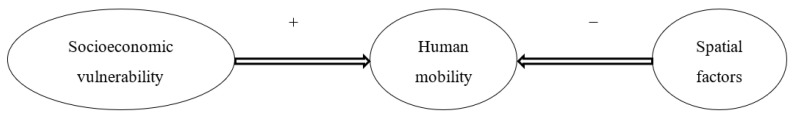
Associations between socioeconomic features, spatial factors, and human mobility.

Collecting travel flow data from 1436 administrative areas of mainland France, researchers in [45] noticed that lockdown reduced short and long-range mobility across the country by 65%. However, an uneven reduction in mobility was observed across people of various socioeconomic backgrounds, geographical locations, and times of the day. A sharper decrease in mobility was reported for the elderly, long-range travel, people employed in the sectors that introduced work-from-home strategies during lockdown periods, and areas with a higher number of hospitalizations and health issues. This study also reported a higher reduction in mobility in major cities due to a higher number of coronavirus cases and stricter lockdown measures. Movements during rush hours were severely affected due to school closure and work-from-home policies. Movements during the daytime on weekends have also been reduced due to a reduction in recreational trips. In contrast, the smallest reduction in movement during nighttime was observed on weekdays due to some mandatory work-related travel. Thus, evidence indicates that socioeconomic and spatial factors and time of day may have a determining impact on human mobility patterns (Figure 6).

### 2.6. Conceptual Framework

**Figure 7:**
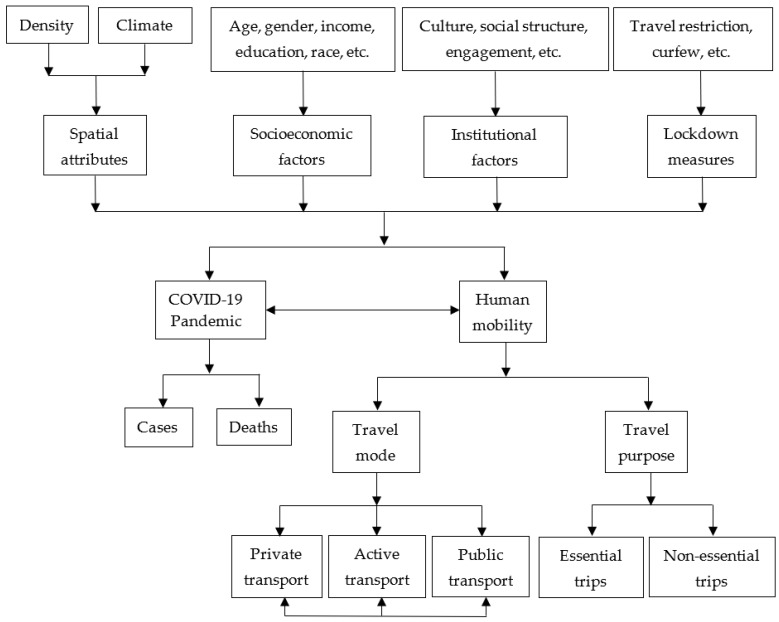
Conceptual framework of the study, modified from [9].

Based on the comprehensive review of the extant literature summarized above, a conceptual framework is developed to study the relationships between lockdown measures, mobility patterns, pandemic severity, and socioeconomic and institutional factors of countries across all world regions (Figure 7). The conceptual framework posits that spatial attributes, socioeconomic factors, institutional contexts, and lockdown and confinements measures are influential considerations in the COVID-19 pandemic and on human mobility. These factors control the severity of the pandemic by increasing or decreasing COVID-19 confirmed cases and deaths. Similarly, they condition human mobility by influencing travel modes and purposes to essential and non-essential places. COVID-19 pandemic and human mobility co-exist through a bi-directional relationship, as they have the potential to influence one another. The framework also postulates that travel modes interact among themselves, which indicates that increasing one mode (e.g., car) automatically reduces demand for other travel modes (e.g., bus). It is used to explicitly model the complex and multifaceted relationships between the COVID-19 pandemic, lockdown and confinement measures, human mobility, socioeconomic, and institutional factors at the granularity of countries drawn from around the globe using structural equation modeling (SEM) techniques. 

## 3. Data and Study Approach

### 3.1. Data

To test and validate the conceptual model internationally at the scale of countries and determine the relative strength of the complex relationships between the factors outlined in Figure 7, data were collected from multiple sources. The data sources include Google, the United Nations (UN), United Nations Development Program (UNDP), Worldometer, Oxford University, Hofstede Insights, The Fraser Institute, KOF Swiss Economic Institute, and BBC. Table 2 contains the list of 19 variables that were included in the final model. However, a complete list of variables that were tested in the SEM framework to achieve the final model was provided in [16]. A complete and consistent dataset was compiled for 86 countries listed in Appendix A. Initially, a total of 131 countries were selected to collect mobility data from Google, considering several important limitations (e.g., the internet is not available in many countries, especially in rural areas, many people usually elderly people do not use smartphones, users must turn on their travel location history). However, a subset of 86 countries was extracted from the list of 131 countries due to inadequate information on socioeconomic attributes, institutional contexts, lockdown measures, and the incidence of the COVID-19 pandemic in the rest of the countries. The analysis reported below is based on this reduced set of countries spanning all major world regions.

Data related to changes in human mobility due to lockdown measures (e.g., travel ban, work-from-home, shelter-in-place, restrictions on public gathering) were collected from Google Mobility Reports [68,75]. This report shows how visits and length of stay at different places, such as retail and recreation (e.g., restaurants, cafés, shopping centers, theme parks), workplaces (i.e., place of work), transit stations (e.g., subway stations, seaports, taxi stands, rest areas), residential areas (i.e., places of residence), parks (e.g., public parks, national forests), grocery stores and pharmacies (e.g., supermarkets, convenience stores, drug stores) changed during the pandemic compared to a baseline value, with a potential to reduce the health outcomes of the COVID-19 pandemic. The baseline value is the median value of the corresponding week during the 5-week period from 3 January to 6 February 2020. This study uses mobility changes in retail and recreation, workplaces, transit stations, and residential areas using the country as units of analysis. Due to the ambiguity in the nature of visits and trips to grocery stores and pharmacies and the inconsistent definition of parks across countries (i.e., only include public parks), mobility changes for these POIs are excluded from the study.

The total daily number of coronavirus infection cases and death cases for the study period were sourced from Our World in Data, a research project at the University of Oxford [70]. They collect data from thousands of sources around the world, analyze and validate them in real-time, and provide country-wise COVID-19 statistics. A 7-day moving average (i.e., adding up the number of cases/deaths for the prior 7 days and dividing the total number by 7) for coronavirus cases and deaths was calculated to take any uninformed cases and deaths on a particular day into account. To flatten the curve of COVID-19, governments issued different lockdown measures for part or a whole country to restrict all non-essential movements. Data related to lockdown measures were collected from Dunford, Dale [69], and Oxford [70]. This study also collected socioeconomic (e.g., age, education, employment sector) and institutional context (e.g., individualism versus collectivism, globalization index) data to investigate their impacts on coronavirus infection cases and deaths, lockdown measures, and travel patterns. 

### 3.2. Study Approach

SEM is used to investigate the causal relationships between socioeconomic and institutional factors, lockdown variables, coronavirus infection and death rates, and social distancing measures and validate the model depicted in Figure 7. This multivariate statistical technique is a common method for investigating complex relationships between dependent variables, independent variables, mediators, and latent dimensions. Many researchers have used SEM to investigate the factors that affect travel behaviors (e.g., mode choice, trip purpose, travel distance), for instance [76,77,78,79]. SEM consists of regression analysis, factor analysis, and path analysis to explore interrelationships among variables. It is a confirmatory technique where a postulated model is tested to check consistency between the existing theories and the nature of constructs.

Based on exploratory factor analysis (EFA) and extant theories reviewed in the previous section of this article, latent dimensions are created to reduce dimensions and easily understand the data and represent underlying concepts. The following four latent dimensions are constructed:

1. Human mobility (i.e., the reverse of Social Distancing): TS, RR, WP, and RD
2. Pandemic severity: l_case and l_death
3. Lockdown measures: NL, WPC, SH, and SI
4. Socioeconomics and institutional factors: MA, AGE65, KOFGI, AE, SE, HE, FS, EI, and IDV.

Moreover, a path diagram is constructed to graphically represent the interdependencies of the independent variables, mediators, and dependent variables in the model specification. Finally, a set of fit indices (e.g., Chi-square, CFI, TLI, RMSEA, SRMR) are estimated to establish the goodness-of-fit of the model. Two models evenly spaced on the timeline are estimated from each month from March 2020 to February 2021, to capture the temporal aspects of the pandemic, how they evolved and influenced the mobility of people, and how people learned and adjusted to the changing environment (Table 3). Hence, a total of 24 models are estimated. For the sake of comparison over time, the same functional specification of the model is estimated for each instance.

## 4. Study Results

**Figure 8:**
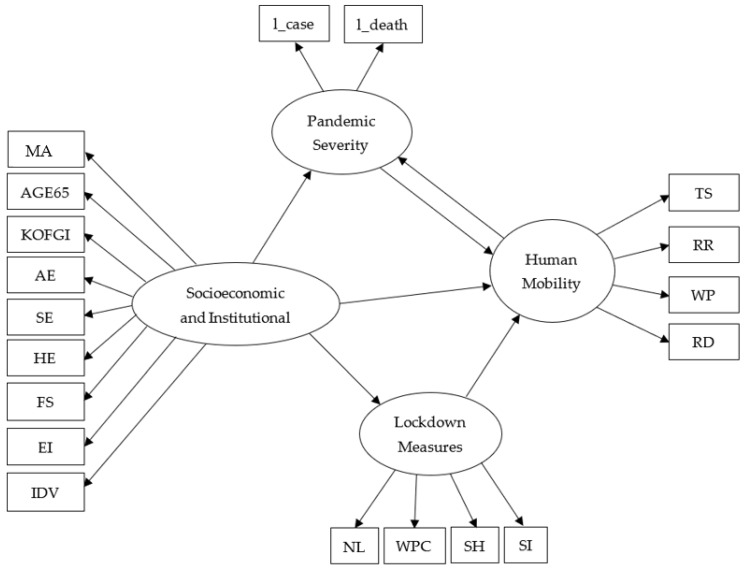
The calibrated model with direct standardized effects.

The models were calibrated using the SEM Builder on the STATA 15 platform [80]. The maximum likelihood estimation method was used to calculate the coefficients. The final structure of the models included interactions between dependent and independent variables through mediators (Figure 8). This figure was used as the basis to estimate the direct and indirect impacts of independent variables on dependent variables and the changes in parameters over time.

The robustness of the calibrated models was assessed with several goodness-of-fit statistics, as presented in Appendix B. The Chi-square (CMIN) statistics of the estimated models ranged from 200 to 400, which were statistically significant. A lower value of CMIN indicates a better fit for the models [16]. The chi-square over the number of degrees of freedom (Chisq/df) with a value of 5 or less is a good measure of model fit [81]. 

The Chisq/df values of all models presented in Appendix B showed a good fit of the models. Similarly, the values of CFI, TLI, and SRMR indicate a good fit of the models with an acceptable range of the values [81,82]. The estimated goodness-of-fit indices demonstrate that the calibrated models matched the actual data as closely as possible. Thus, by all means, the performance of the models was quite satisfactory, which validated the usefulness of the analysis and of the models that were generated. 

### 4.1. Impacts of Lockdown and Confinement Measures

#### 4.1.1. Impacts on Human Mobility

**Figure 9:**
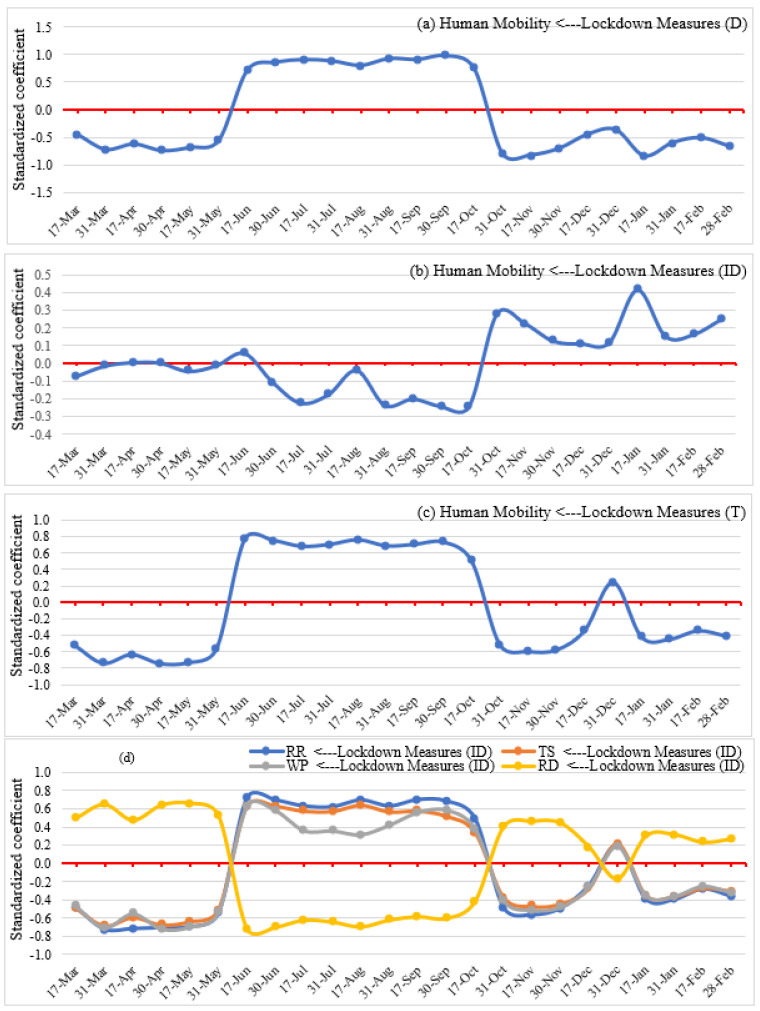
Direct (**a**), indirect (**b**,**d**), and total (**c**) effects of lockdown measures on human mobility.

The direct, indirect, and total effects of lockdown and confinement measures on human mobility (i.e., the reverse of social distancing) from 17 March 2020 to 28 February 2021 are depicted in Figure 9. Direct impacts of lockdown measures (Figure 9a) show that, at the onset of the pandemic (March to mid-May), governments that adopted lockdown and confinement measures significantly enhanced the practice of social distancing and reduced human mobility in their jurisdiction (i.e., a negative association between lockdown measures and mobility). People appear to have fairly strictly abided by the social distancing measures induced by the fear of COVID-19 infection, which resulted in sharply reduced mobility of people. However, human mobility increased from mid-May to mid-October (i.e., positive association) compared to the baseline value (i.e., the 5 weeks from 3 January to 6 February 2020) in response to somewhat relaxed lockdown measures and people’s fatigue with government decrees and their yearning for a return to normal life. Consequently, a sudden rise in COVID-19 cases and related deaths in most countries were observed, with a peak from November 2020 to mid-January 2021. Considering the renewed vigor of the pandemic during this time frame and reinstated NPIs, human mobility further reduced significantly in most countries.

In addition to their direct effect on social distancing patterns, lockdown measures can also have an indirect impact (Figure 8) on human mobility patterns through the feedback intermediation of the viral spread itself. Figure 9b indicates that this overall indirect impact was, in fact, very limited and rather insignificant throughout the study period, even offsetting to some extent the direct effects starting in June 2020. Thus, a decreasing trend (i.e., negative association) was observed from mid-June to mid-October due to relaxed lockdown measures and the manageable state of the pandemic. The reverse happened from mid-October to February 2021 due to the severity of the pandemic and people’s self-quarantine to protect themselves and family members from the COVID-19-related health risks. 

The impacts of lockdown and confinement measures on each of the factors of human mobility patterns (i.e., human activity at retail and recreation sites, transit stations, workplaces, and residential areas) can be estimated by the same token (Figure 9d). Overall, they closely track the total impacts of lockdown. Human activity in residential areas was higher compared to the baseline scenario from March to mid-May and mixed with fluctuations from mid-October to February. In contrast, between mid-May and mid-October, people sought to escape stay-at-home directives. Understandably, compared to residential areas, human activity in retail and recreation, transit stations, and workplaces exhibited the reverse response to lockdown measures. They dropped from March to mid-May and from mid-October to February (with fluctuations during the year’s end holiday season) and increased from mid-May to mid-October. The nature of impacts of lockdown measures on social distancing and human mobility at various POIs explains that with the increasing strictness of lockdown measures and the severity of the pandemic, people maintained social distancing practices and thereby stayed at home; they strived to avoid essential travel to retail and recreation, transit stations, and workplaces. In contrast, people responded to the relaxation of lockdown measures by overcompensating with travel to retail and recreation facilities, transit stations, and workplaces.

Considering the direct and indirect effects discussed so far, Figure 9c depicts the total impacts of lockdown and confinement measures on human mobility patterns. These impacts overwhelmingly reflect direct effects. Specifically, the figure indicates notably diminished human mobility between March and mid-May as a result of lockdown measures, and the same also transpires for the period from mid-October to February, although the effect is weaker and even opposite in late December, possibly as a result of lower behavioral compliance during the end of the year holiday season. In contrast, a significant increase in human mobility was observed from mid-May to mid-October as the first wave of infection eased in many countries. Overall, the figure explains that people practiced social distancing when the government imposed strict lockdown measures, which therefore reduced mandatory and non-mandatory trips. On the other hand, the tendency to maintain social distancing reduces when authorities ease lockdown measures, and people want a break from monotonous daily living. Thus, temporal changes in social distancing patterns are observed with adjustments to the strictness of lockdown measures and the severity of the pandemic at different time points (e.g., March to mid-May, mid-May to mid-October, and mid-October to February) throughout the study period.

#### 4.1.2. Impacts on COVID-19 Pandemic

**Figure 10:**
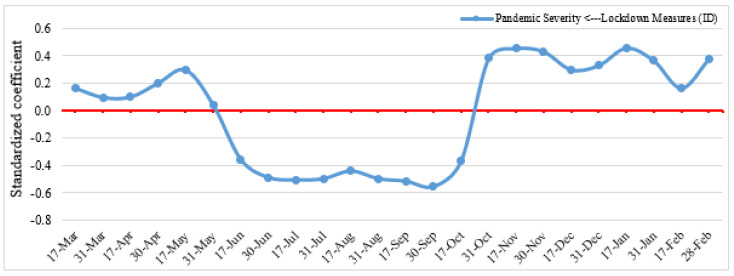
Indirect effects of lockdown measures on pandemic severity.

The impacts of COVID-19-related lockdown and confinement measures on overall pandemic severity, COVID-19 confirmed cases, and deaths, are presented in Figure 10. The 24 models calibrated over the study period found no direct impact of lockdown measures on the COVID-19 pandemic. However, an indirect impact is portrayed, with the mediating effect of social distancing/human mobility patterns. Specifically, evidence shows that the pandemic reached greater severity (i.e., positive association), including COVID-19 confirmed cases and deaths, in countries that had enacted lockdowns from March to May and mid-October to February. The pandemic was also found to have eased out (i.e., negative association) in locked-down countries compared to others from June to mid-October. What may explain these contrasted responses is that, during the latter months, lockdown measures received greater compliance from populations, particularly those in countries that were afflicted by a higher incidence of the pandemic, which resulted in more social distancing and a sharp reduction in human mobility at various POIs. During the early months of the pandemic, social distancing was rather unevenly practiced as people were still learning about the pandemic and ways to effectively mitigate it, messaging from public health officials was often lacking consistency, and people also willfully ignored government directives. After October, as the pandemic somewhat eased out, remaining lockdown measures were increasingly seen as oppressive of people’s freedoms and disregarded by populations eager to return to more normal daily lives. Thus, it can be concluded that lockdown measures influenced the severity of the pandemic by moderating factors of social distancing practices, but human factors of learning, uncertainty, fatigue, and contempt and defiance towards government downgraded their effectiveness. 

### 4.2. Impact of Pandemic Severity on Human Mobility

**Figure 11:**
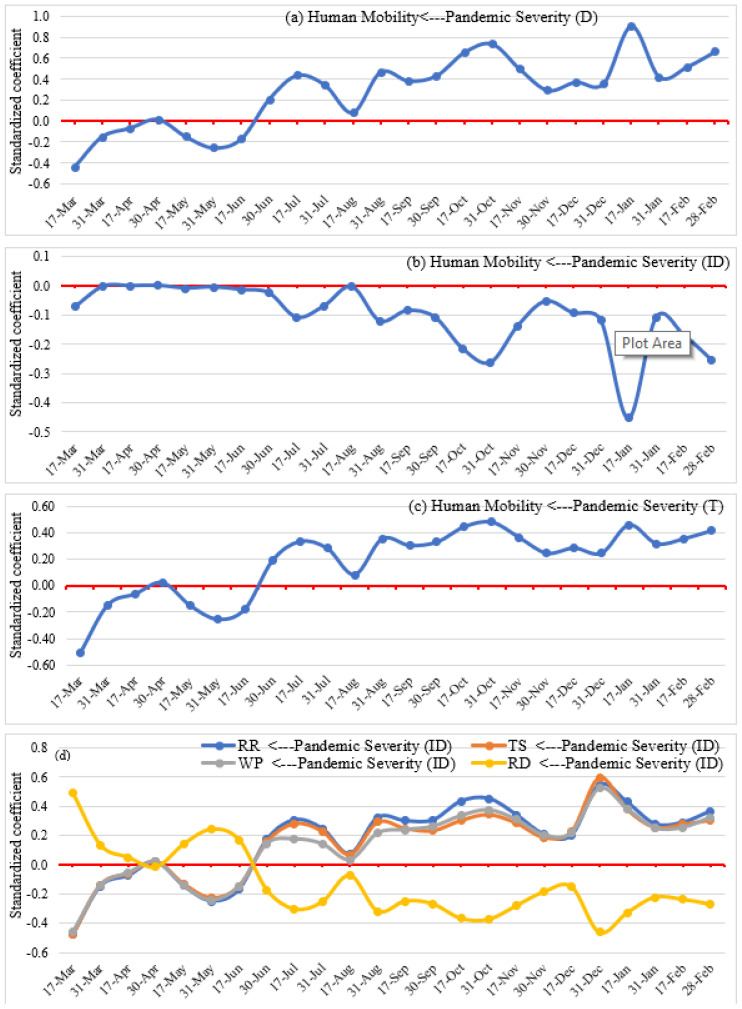
Direct (**a**), indirect (**b**,**d**), and total (**c**) effects of pandemic severity on human mobility.

The direct, indirect, and total effects of the pandemic on human mobility over the study period are captured in Figure 11. There is a negative direct association between pandemic severity and social distancing from March to mid-June, which means that countries where the pandemic was raging experienced reduced human mobility at retail and recreation facilities, transit stations, and workplaces and increased time spent at residences. However, the association was statistically not very significant nor strong. The rest of the pandemic is in sharp contrast, with the pandemic leading directly to lower social distancing and more mobility from mid-June to February, although fluctuations in the magnitude of the response are well discernable. It can be conjectured that, after months of strict lockdown measures and associated social and economic disruptions, and the trepidations of the early phase of the pandemic, people and business organizations were desperate to go back to normal life and resume usual pre-pandemic activities. Consequently, whether or not authorities relaxed the strictness of different lockdown measures, higher human mobility at different POIs ensued, regardless of the increasing pandemic severity.

The indirect impact (Figure 11b) of the pandemic on human mobility is marked by self-moderation. The figure presents a relatively weak negative indirect association between pandemic severity and mobility, which indicates that during the peak time of the pandemic, people self-regulated their travel demands and stayed at home due to the fear of COVID-19 infection regardless of relaxed lockdown measures.

Drilling down deeper into human engagement in various activities, Figure 11d shows the indirect impacts of pandemic severity on human mobility at retail and recreation facilities, transit stations, workplaces, and residential areas. Specifically, pandemic severity negatively influenced human activity at retail and recreation facilities, transit stations, and workplaces while leading to more time spent home from March to mid-June. The reverse is observed from mid-June to February. Thus, during the early phase of the pandemic, people reduced their movement to different POIs due to lockdown measures and self-awareness induced by fear of infection; however, at the later stage, people started to travel for different purposes, considering the social and economic loss, prolonged nature of the pandemic, and relaxed lockdown measures. 

Combining direct and indirect impacts, Figure 11c reveals the total impacts of the pandemic on mobility. We find a trend of associations that largely mirrors the direct effects discussed above (i.e., negative association from March to June and positive association from June to February). Thus, overall, the human toll of the pandemic situation fails to motivate people to curb their mobility when the crisis survives for a long time. Human behaviors are complex and cannot readily be brought to a single dimension. Whereas the model controls for a number of other factors and influences, it is clear the contextual complexity of individual and social responses with social distancing continues to defy simple understandings, particularly given the heterogeneous contexts of countries [9,22,83]. 

### 4.3. Impacts of Human Mobility on the Severity of the Pandemic

**Figure 12:**
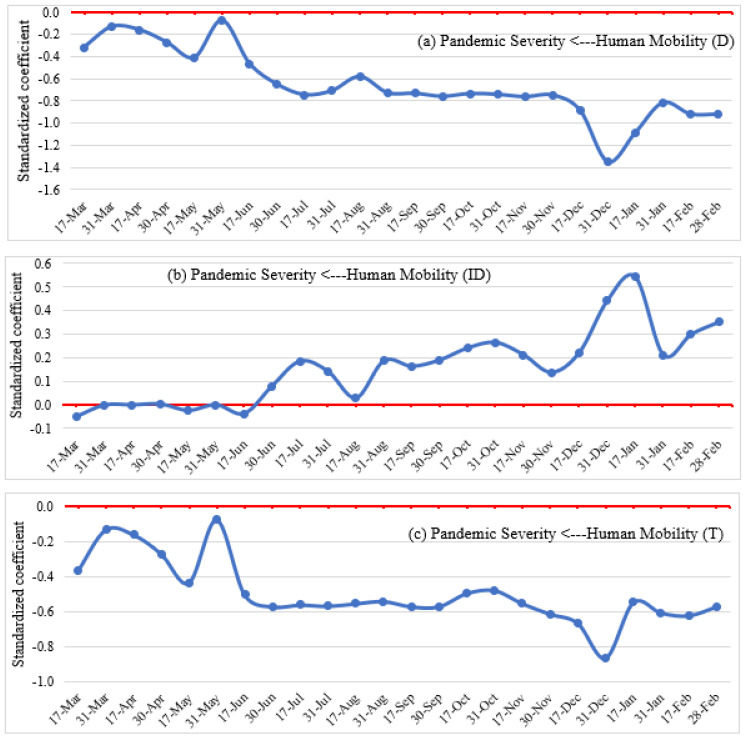
Direct (**a**), indirect (**b**), and total (**c**) effects of human mobility on pandemic severity.

Figure 12 depicts the direct, indirect, and total effects of human mobility (and, therefore, social distancing practices) on pandemic severity over the study period. The SEM analysis isolated a strong negative direct impact (Figure 12a) of social distancing on pandemic severity throughout the study period compared to the baseline scenario. This effect was greatest in late December 2020 and early January 2021, in fact, in the order of 3 to 4 times larger than in the early weeks of the pandemic (between March and May 2020). Thus, the analysis shows that countries that have reduced their personal mobility more have experienced a deepening of the severity of the pandemic (i.e., more infection cases and more deaths). This, of course, may strike as counterintuitive. However, it is worth restating here that this effect is observed while controlling for the role of lockdown measures enacted in a number of countries, or for that matter, the lack thereof. In our model, the effect of lockdown measures is captured separately, as discussed in Section 4.1.2.

The indirect impact (Figure 12b) of human mobility on pandemic severity happens by self-moderation. It was found to be much smaller than its direct counterpart, and hovered around zero from March to mid-June. In contrast, a wavering positive impact is observed from mid-June to February, with a relatively significant impact from December due to the self-regulated effect. During this time, people reduced social distancing and increased essential and non-essential trips due to relaxed lockdown measures and a somewhat open economy, which substantially increased the severity of the pandemic.

Combining direct and indirect impacts, the overall trend (Figure 12c) shows the preponderance of the direct effects on the severity of the pandemic. Specifically, in and of itself, the reduction of personal mobility in countries, in fact, led to a deepening of the pandemic, in spite of the self-moderating effect noted earlier, assuming away all other factors, especially the enactment of lockdown measures by various levels of governmental authorities. Therefore, we find that home confinement enhances the risk of infection from other members of the household, if other NPIs are not adequately implemented and the stringency of enforcement aimed at curtailing community infection is not accounted for.

### 4.4. Impacts of Socioeconomic and Institutional Factors

#### 4.4.1. Impacts on Human Mobility

**Figure 13:**
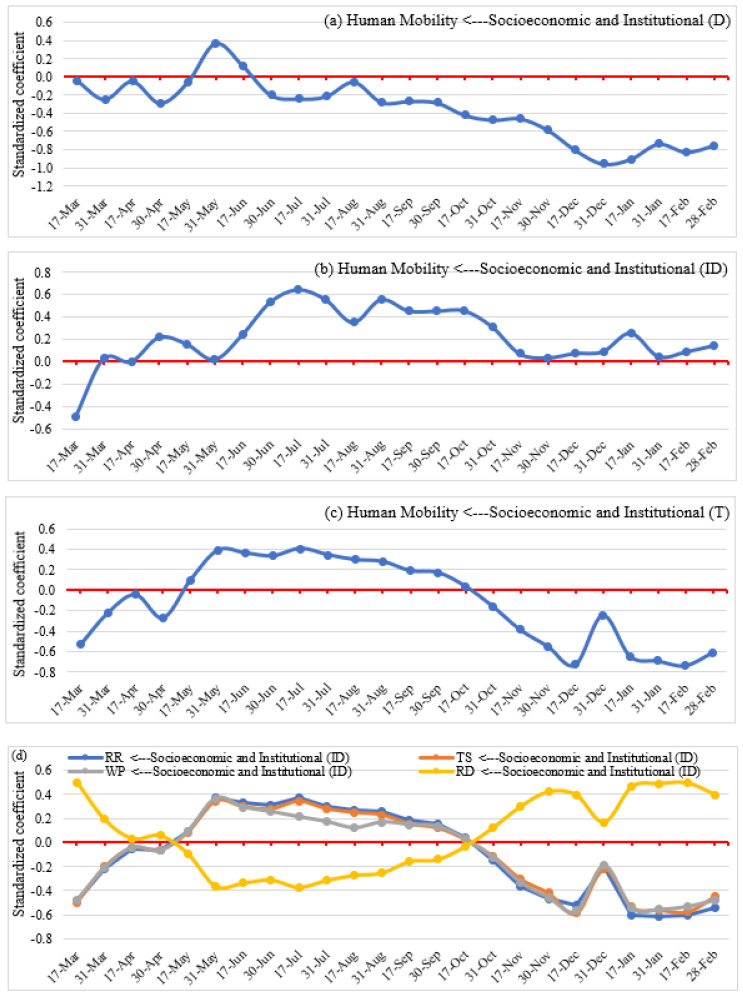
Direct (**a**), indirect (**b**,**d**), and total (**c**) effects of socioeconomic and institutional factors on human mobility.

The direct, indirect, and total effects of socioeconomic and institutional factors on social distancing (i.e., human mobility) are presented in Figure 13. Overall, socioeconomic and institutional factors have negative direct associations with mobility throughout the study period, with a positive association for a short time only (i.e., from mid-May to mid-June). These effects were also found to be deepening as the pandemic went on. Thus, socioeconomic and institutional factors provide a context that encourages people to maintain social distancing (i.e., reduction in necessary and unnecessary travel to POIs) considering the greater vulnerability of the elderly, higher treatment costs, higher level of globalization, increasing employment in the service sector, and decreasing employment in the agricultural sector, degree of independence in society.

The indirect effects (Figure 13b) of socioeconomic and institutional factors on mobility are moderated by the pandemic severity and lockdown measures (Figure 8). Except for the month of March 2020, these effects are positive. Through complex mediations, socioeconomic and institutional conditions indirectly encourage people to be more mobile by enabling the pandemic to reach higher levels of acuity and enabling stricter lockdown measures, considering the greater risk of infection due to fragile public health conditions, higher risk of infection from globalization, and open interactions. 

The total impact of socioeconomic and institutional factors on social distancing measures (Figure 13c) shows an increase in human mobility from mid-May to mid-October to February and a negative effect before and after that period. Thus, we find that socioeconomic and institutional factors may encourage people to maintain social distancing or lead to greater personal mobility at different stages of the pandemic. In particular, we see that social distancing was the norm internationally earlier on, then went away for several months before being espoused quite assiduously in the Fall and Winter of 2020 and 2021.

#### 4.4.2. Impacts on Pandemic Severity

**Figure 14:**
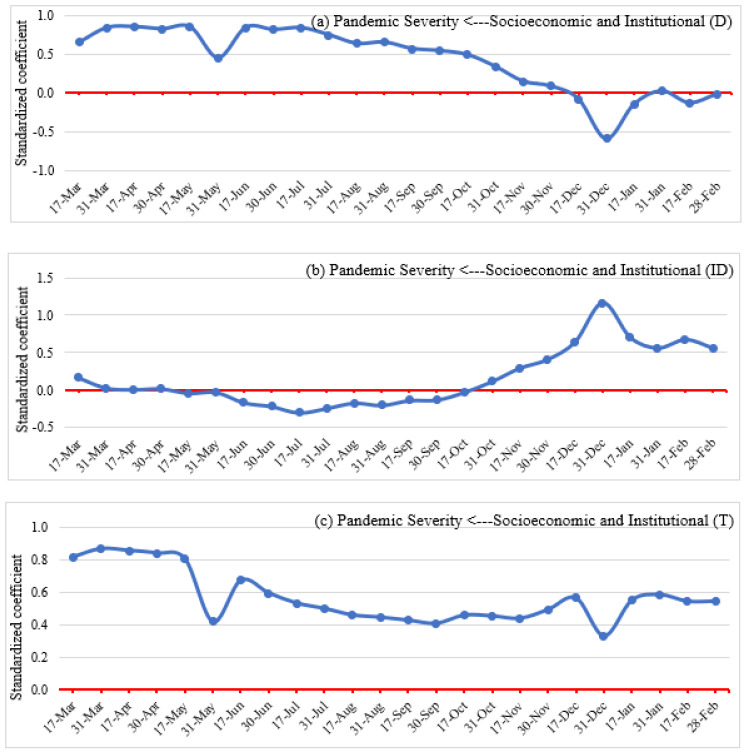
Direct (**a**), indirect (**b**), and total (**c**) effects of socioeconomic and institutional factors on pandemic severity.

The direct, indirect, and total effects of socioeconomic and institutional factors on pandemic severity are shown in Figure 14. The direct impacts (Figure 14a) were generally positive, despite showing a negative association at the end of the study period (i.e., mid-December to February). Thus, socioeconomic and institutional factors increased the severity of the pandemic due to a higher number of elderly people who were physically susceptible to COVID-19 infection, a higher level of globalization, higher employment in the service sector and lower employment in agriculture, and to a higher degree of personal independence in society.

Indirect effects (Figure 14b) of socioeconomic and institutional factors on the severity of the pandemic are moderated by lockdown measures and social distancing patterns. SEM results show a negative and relative insignificant negative indirect impact of socioeconomic and institutional circumstances from March to mid-October 2020 due to implemented lockdown measures and people’s tendency to maintain social distancing. However, a positive and strong association is observed from mid-October to February due to a slight relaxation of lockdown measures, people’s intention to go back to normal life, and increasing human mobility to different POIs. 

The overall impacts (Figure 14c) of socioeconomic and institutional factors on pandemic severity involve a positive association of socioeconomic and institutional factors and pandemic severity all through the study period. Thus, socioeconomic and institutional factors increase COVID-19 confirmed cases and deaths via a larger elderly population, higher health costs, deeper involvement in globalization, more employment in the service sector, and less employment in agriculture. 

## 5. Discussion

Considering the direct and indirect effects and their temporal variation, this study reported a negative association between lockdown measures and human mobility at the onfall of the pandemic from March 2020 to mid-May 2020. People maintained strong social distancing (i.e., lower tendency to travel for essential and non-essential purposes) when the government imposed strict lockdown measures and restrictions on mobility. On the other hand, citizens defied social distancing practices when authorities eased lockdown measures between mid-May and mid-October 2020, and they aspired to go back to normal life and break the dullness of their confined life. However, a further reduction in human mobility was observed from November 2020 to mid-January 2021 due to the reinstatement of lockdown measures induced by the sharp rise in coronavirus cases and deaths. Thus, strict lockdown measures significantly reduced human mobility across countries, which upholds the findings from our previous study [16], where we showed that strict lockdown measures substantially decreases human mobility at retail and recreation facilities, transit station, and workplaces and encourage people to stay home and avoid unnecessary travel. Similarly, researchers in [84] reported a significant drop in human mobility after introducing lockdown in Chinese cities. Conducting a study in 15 European cities, Santamaria, Sermi [85] observed a reduction in human mobility during the lockdown periods and an increase in human mobility after effectively lifting the lockdown and confinement measures. Moreover, they mentioned that lockdown and confinement measures could explain up to 90% of the variability in mobility patterns. Thus, lockdown and confinement measures are very effective in reducing human mobility for controlling the diffusion of a viral infection such as COVID-19.

We have also shown that lockdown and confinement measures indirectly influence the COVID-19 pandemic by moderating social distancing/human mobility patterns. The study findings indicate that the pandemic severity intensified in some countries from March to May due to the inadequate practice of social distancing and from mid-October to February due to the increase in human mobility induced by relaxed lockdown measures and people’s eagerness to return to normal life. However, the study noticed that adequate and timely adopted lockdown measures reduced the severity of the pandemic from June to mid-October, which corroborates the findings from previous studies [16,86,87,88]. Study findings in [89] reported that restrictions on human mobility and person-to-person contact reduce COVID-19 transmission by 45%. However, delayed lockdown measures and people’s unwillingness to maintain social distancing, and their return to traveling to retail and recreation facilities, transit stations, and workplaces can increase the severity of the pandemic. Thus, researchers in [86] suggested evaluating mobility changes after the implementation of lockdown measures along with appropriate messaging and announcement (i.e., safe preparation, the burden on the supply chain) and resource allocation to prevent any unintended consequences.

Socioeconomic and institutional conditions encourage people to practice social distancing (i.e., mobility reduction), considering the greater vulnerability of the elderly, higher treatment costs, higher level of globalization, increasing employment in the service sector, decreasing employment in agriculture, and the degree of independence in national societies. These factors also motivate people to maintain social distancing while the pandemic is more severe and lockdown measures are more strict, considering the greater risk of COVID-19 infection due to fragile public health conditions, higher risk of infection from globalization, and open interactions. Thus, socioeconomic and institutional factors have significant impacts on maintaining social distancing to reduce the severity of the pandemic [16,90,91].

This study also found a significant impact of socioeconomic and institutional factors on the severity of the pandemic. Considering direct and indirect impacts, we observed that socioeconomic and institutional factors increased the severity of the pandemic due to a higher number of elderly people who are physically susceptible to COVID-19, a higher level of globalization, increasing employment in the service sector, a reduction in agriculture, and a higher degree of independence in society. Thus, socioeconomic and institutional factors increase the severity of a pandemic by reducing social distancing practices and increasing human mobility for essential and non-essential purposes [16,90,91,92].

The study found mixed effects of the COVID-19 pandemic (i.e., negative and positive) on maintaining social distancing. The negative association at the early stage of the pandemic (March to June) indicates that the increasing severity of the pandemic reduced human mobility and increased time spent at their residence. Early on, people reduced their movement due to lockdown measures and self-awareness induced by fear of infection, which endorses the findings from previous studies [16,26,93,94]. However, the association was not statistically very significant and robust. In contrast, a positive association from mid-June to February indicates that human mobility increases despite the increasing severity of the pandemic. This can be ascribed to people and business organizations becoming desperate to go back to normal life and resume usual daily activities after prolonged and strict lockdown measures and associated social and economic disruptions. Consequently, authorities relaxed the stringency of lockdown measures, which induced higher human mobility regardless of increasing pandemic severity. However, overall, pandemic situations have little influence on reducing human mobility when the crisis is sustained for a long time. 

Investigating the impact of human mobility on pandemic severity, we observed a strong negative and sustained impact of mobility/social distancing on pandemic severity throughout the study period compared to the baseline scenario, with the greatest impacts in late December 2020 and early January 2021. The study showed that countries with a sharper reduction in personal mobility had experienced an increased severity of the pandemic due to the absence of other NPIs and the possibility of infection from other family members while home confinement. Thus, maintaining social distance lessens the severity of the pandemic (i.e., the number of infection cases and deaths) by reducing human trips to retail and recreations, transit stations, and workplaces and encouraging people to stay at home, which confirms the results from previous studies [16,95,96]. However, people reduced social distancing and increased essential and non-essential trips due to relaxed lockdown measures and the desire to open the economy, which substantially increased human mobility and the severity of the pandemic. Conducting a study in European countries, researchers in [97] mentioned that human mobility is solely responsible for up to 92% of the early diffusion of COVID-19, although a declining trend is reported after implementing lockdown measures and restrictions on mobility. Thus, appropriate timing and practicing social distancing is one of the key factors in controlling the outbreak of the pandemic [98].

## 6. Conclusions and Directions for Future Research

Given the adverse effects of the COVID-19 pandemic, there is a pressing need to understand the root causes of the pandemic, associated risks, and conditions that exacerbate its impacts and promising mitigation measures. This study intended to perform a longitudinal study spanning a great diversity of countries, with data from March 2020 to February 2021. The main objective of this longitudinal study was to explore the evolution of complex relationships between the incidence of the COVID-19 pandemic, lockdown measures on populations, and their social distancing and mobility behaviors in 86 countries. A conceptual framework was developed (Figure 7) to hypothesize the spatial attributes, socioeconomic factors, institutional contexts, and lockdown and confinements measures that would influence the COVID-19 pandemic and human mobility. To test and validate the conceptual model, an SEM framework was applied to calibrate 24 models on data collected from multiple sources (Table 2) to test and validate the conceptual model.

The study results indicate that implementation of lockdown and confinement measures are necessary to reduce human mobility and encourage people to stay home to control COVID-19 transmission through community infection. However, delayed implementation of lockdown measures, relaxation of lockdown measures, and people’s failure to maintain social distancing and their travel for work and discretionary activities can worsen the pandemic. Thus, it is suggested to evaluate mobility changes after the implementation of lockdown measures, which can guide policymakers on when to implement stricter lockdown measures or ease them, to curtail the diffusion of coronavirus. The study also found that national context defined by socioeconomic and institutional factors influences social distancing patterns and the severity of the pandemic. Additionally, this study observed an intertwined two-way association between the COVID-19 pandemic and human mobility. Higher severity of the pandemic reduces human mobility and on the other hand, higher human mobility increases the severity of the pandemic. However, the impact of human mobility on pandemic severity is stronger and more significant compared to the reverse relationship. Overall, the study displays considerable temporal changes in the relationships between independent variables, mediators, and dependent variables considering pandemic situations and lockdown regimes.

Several policy decisions have been outlined based on the analysis. The timely implementation of strict and comprehensive lockdown and confinement measures is critical to mitigating the severity of the pandemic by reducing the movement of people and person-to-person interactions. Moreover, estimating the changes in human mobility during lockdown periods is an effective policy option to evaluate the social distancing patterns of people. If necessary, a targeted campaign among the less responsive communities or individuals could be effective in enhancing social distancing practice, in addition to vaccine administration at a large scale [99,100]. Since mass transportation is risky during pandemic situations, micro-mobility (e.g., e-scooter, cycling) could be promoted to facilitate safer travel of people [9]. 

Although this study has revealed the changing faces of the pandemic and provides significant policy implications, adopting an SEM using actual longitudinal data (i.e., panel data) and showing temporal effects in path diagrams could reveal complex causal relationships between COVID-19 pandemic, lockdown measures, and human mobility [101]. Since mobility changes in transit stops do not necessarily represent the condition of transportation systems and infrastructure for public transportation, future studies should consider the effects of public transport infrastructure, social distancing regulations inside the public transportation system, and the governance of public transportation on human mobility and COVID-19 pandemic. Moreover, the application of machine learning-based models may offer additional insights to control this multifaceted problem. 

The goodness-of-fit statistics of the models (Appendix B) showed a deviation from the suggested cut-off values, which may raise concerns about the statistical significance of our results. However, it is a standard practice held by applied researchers as long as an adequate scientific explanation is provided and the results are grounded in and consistent with the extant literature and theories, even if there is a small deviation from the suggested cut-off values. Here are the main points that guide us in making decisions on the fit and significance of our results, given that the number of independent countries in not subject to sampling. First, a simulation study [102] found that the values of fit indices are very sensitive to sample size, factor loadings, and to the number of indicators. The study suggested adjusting the fit indices for the effects of sample size and factor loadings before comparing them with arbitrary cut-off values. Second, fit indices are one of the many methods to assess the goodness-of-fit of the model. Thus, researchers have strongly suggested consulting extant theories to assess the fit of the models rather than solely relying on the fit indices derived from empirical methods [103]. Third, triggered by the uncertainty and contextual value of fit indices to meet the recommended cut-off points, researchers characterize the validity of model findings based on their scientific understanding [104]. 

## Figures and Tables

**Table 1:** Changes in personal modal choices during lockdown regimes, adopted from [23].

Transport Mode	United States	Europe	China
Public transit (metro, bus)	−60% or above	−60% or above	−60% or above
Ride-hailing (on-demand, pooled)	−60% or above	−60% or above	−60% or above
Ride-hailing (on-demand, single)	−60% or above	−60% or above	−60% or above
Car sharing (free-floating or station-based)	−60% or above	−60% or above	−60% or above
Bike sharing (free-floating or station-based)	+21% to +59%	−21% to −59%	+21% to +59%
Scooter sharing	−60% or above	−60% or above	N/A
Bike/e-scooter, walking	+21% to +59%	+21% to +59%	+21% to +59%
Private car (own or company supported)	−21% to −59%	−60% or above	−21% to −59%

**Table 2:** Description of the variables and data sources.

Variable	Description	Source
RR	Percentage change of mobility in retail and recreation trips	[68]
TS	Percentage change of mobility in transit stations trips	[68]
WP	Percentage change of mobility in workplaces trips	[68]
RD	Percentage change of mobility in residential trips	[68]
l_case	Total coronavirus infection cases per 1 million population (log transform)	[1]
l_death	Total coronavirus deaths per 1 million population (log transform)	[1]
NL	National lockdown	[69]
WPC	Workplace closing	[70]
SH	Stay-at-home order	[70]
SI	Stringency index ^i^	[70]
FS	Percentage of female smokers	[70]
AGE65	Percentage of the population aged 65 and older	[70]
MA	Median age	[71]
EI	Average of years of schooling vs. expected years of schooling	[71]
AE	Percentage of the population employed in agriculture	[72]
SE	Percentage of the population employed in services	[72]
HE	Percentage of health expenditure to total GDP	[72]
IDV	Individualism versus Collectivism emphasis ^ii^	[73]
KOFGI	KOF Globalization Index ^iii^	[74]

^i^ A composite index considering all implemented lockdown measures. The score of this index ranges from 0 to 100. A high score indicates the strictest measures and low score indicates loose measure. ^ii^ This indicator measures the degree of interdependence among the members of a society. The score ranges from 0 to 100. A low score indicates collective culture and higher interdependence among the members and conversely a high score indicates Individualist culture and a low level of interdependence. ^iii^ A composite index that indicates openness to trade and capital flows considering economic, social and political aspects. The score of the index ranges from 0 to 100. A high score denotes a highly globalized country and a low score indicates poorly globalized country.

**Table 3:** Models estimated in this study.

S.N.	Models	Dates
1	1 and 2	17 March and 31 March 2020
2	3 and 4	17 April and 30 April 2020
3	5 and 6	17 May and 31 May 2020
4	7 and 8	17 June and 30 June 2020
5	9 and 10	17 July and 31 July 2020
6	11 and 12	17 August and 31 August 2020
7	13 and 14	17 September and 30 September 2020
8	15 and 16	17 October and 31 October 2020
9	17 and 18	17 November and 30 November 2020
10	19 and 20	17 December and 31 December 2020
11	21 and 22	17 January and 31 January 2021
12	23 and 24	17 February and 28 February 2021

## Data Availability

The data presented in this study are available on request from the corresponding author.

## Appendix A

**Table A1:** The list of 86 countries used in the analysis.

SN	Country	SN	Country
1	Angola	44	Libya
2	Argentina	45	Lithuania
3	Australia	46	Luxembourg
4	Austria	47	Malaysia
5	Bangladesh	48	Malta
6	Belgium	49	Mexico
7	Brazil	50	Mozambique
8	Bulgaria	51	Namibia
9	Burkina Faso	52	Nepal
10	Canada	53	Netherlands
11	Cape Verde	54	New Zealand
12	Chile	55	Nigeria
13	Costa Rica	56	Norway
14	Croatia	57	Pakistan
15	Czech Republic	58	Panama
16	Denmark	59	Peru
17	Dominican Republic	60	Philippines
18	Ecuador	61	Poland
19	Egypt	62	Portugal
20	El Salvador	63	Qatar
21	Estonia	64	Romania
22	Fiji	65	Saudi Arabia
23	Finland	66	Senegal
24	France	67	Singapore
25	Germany	68	Slovakia
26	Ghana	69	Slovenia
27	Greece	70	South Africa
28	Guatemala	71	South Korea
29	Honduras	72	Spain
30	Hungary	73	Sri Lanka
31	India	74	Sweden
32	Indonesia	75	Switzerland
33	Iraq	76	Tanzania
34	Ireland	77	Thailand
35	Israel	78	Trinidad and Tobago
36	Italy	79	Turkey
37	Jamaica	80	United Arab Emirates
38	Japan	81	United Kingdom
39	Jordan	82	United States
40	Kenya	83	Uruguay
41	Kuwait	84	Venezuela
42	Latvia	85	Vietnam
43	Lebanon	86	Zambia

## Appendix B

**Table A2:** Goodness-of-fit statistics of the models.

Model #	Chi-Square	Chisq/df	RMSEA	CFI	TLI	SRMR
1	201.120	1.559	0.081	0.958	0.945	0.076
2	289.406	2.243	0.120	0.915	0.887	0.118
3	261.331	2.026	0.108	0.920	0.894	0.099
4	322.505	2.500	0.132	0.893	0.859	0.127
5	391.566	3.035	0.154	0.857	0.810	0.153
6	372.752	2.890	0.148	0.865	0.821	0.177
7	353.937	2.743	0.142	0.872	0.831	0.150
8	317.053	2.458	0.130	0.892	0.857	0.110
9	280.169	2.172	0.117	0.911	0.882	0.131
10	281.313	2.181	0.117	0.907	0.876	0.127
11	280.169	2.172	0.117	0.911	0.882	0.131
12	302.100	2.342	0.125	0.894	0.860	0.123
13	307.364	2.383	0.127	0.892	0.857	0.119
14	280.600	2.175	0.117	0.905	0.874	0.107
15	253.788	1.967	0.106	0.917	0.890	0.117
16	303.425	2.352	0.125	0.884	0.847	0.113
17	261.692	2.029	0.109	0.917	0.890	0.098
18	281.006	2.178	0.117	0.904	0.873	0.095
19	278.128	2.156	0.116	0.903	0.872	0.096
20	280.201	2.172	0.117	0.910	0.881	0.087
21	258.293	2.002	0.108	0.922	0.897	0.090
22	328.738	2.548	0.134	0.881	0.843	0.095
23	258.864	2.007	0.108	0.918	0.891	0.093
24	247.352	1.917	0.103	0.925	0.900	0.093

RMSEA (root mean squared error of approximation), CFI (comparative fit index), TLI Tucker–Lewis index), SRMR (standardized root mean squared residual).
